# Resident Alveolar Macrophages Are Susceptible to and Permissive of *Coxiella burnetii* Infection

**DOI:** 10.1371/journal.pone.0051941

**Published:** 2012-12-19

**Authors:** Matthew Calverley, Sara Erickson, Amanda J. Read, Allen G. Harmsen

**Affiliations:** 1 Department of Immunology and Infectious Diseases, Montana State University, Bozeman, Montana, United States of America; 2 Office of the Senior Associate Vice President for Research, The University of Texas at San Antonio, San Antonio, Texas, United States of America; University of São Paulo, Brazil

## Abstract

*Coxiella burnetii*, the causative agent of Q fever, is a zoonotic disease with potentially life-threatening complications in humans. Inhalation of low doses of *Coxiella* bacteria can result in infection of the host alveolar macrophage (AM). However, it is not known whether a subset of AMs within the heterogeneous population of macrophages in the infected lung is particularly susceptible to infection. We have found that lower doses of both phase I and phase II Nine Mile *C. burnetii* multiply and are less readily cleared from the lungs of mice compared to higher infectious doses. We have additionally identified AM resident within the lung prior to and shortly following infection, opposed to newly recruited monocytes entering the lung during infection, as being most susceptible to infection. These resident cells remain infected up to twelve days after the onset of infection, serving as a permissive niche for the maintenance of bacterial infection. A subset of infected resident AMs undergo a distinguishing phenotypic change during the progression of infection exhibiting an increase in surface integrin CD11b expression and continued expression of the surface integrin CD11c. The low rate of phase I and II Nine Mile *C. burnetii* growth in murine lungs may be a direct result of the limited size of the susceptible resident AM cell population.

## Introduction

*Coxiella burnetii*, an NIAID category B priority pathogen and select agent, is the bacterial pathogen responsible for the clincal symptoms of the zoonotic disease Q fever. In humans, this febrile disease is frequently acquired via inhalation and is characterized by a flu-like illness which can progresss to pneumonia with severe complications resulting from occurrences of chronic infection. Various strains of *C. burnetii* are recognized. Strain designation was historically determined by either geographical location or clinical isolation of bacterial variants. However, molecular methods have determined that strains are also separated by unique grouping characteristics at the genetic level, such as restriction length polymorphism and plasmid content. Yet it was also noted in these studies that this genetic variation does not directly correlate to virulence; therefore, it was suggested that virulence may be more directly attributable to host response [1], [2]. Bacterial phase variants are also recognized irrespective of strain type. Phase I *C. burnetii* is characterized by the presence of O-antigen in the bacterial LPS coat. After serial passage, in egg-yolk or cell culture, gene deletion may occur leading to loss of O-antigen, resulting in the formation of phase II *C. burnetii* [3], [4].

In light of the potential role in virulence played by host response to infection, it is critically important to gain an understanding of early immune responses to infection. Despite this need, a clear understanding of the host innate immune response to pulmonary infections with *C. burnetii* has, thus far, been lacking. The macrophage is the cell-type most responsible for harboring *C. burnetii* and facilitating replication within a parasitophorous vacuole [5]. Moreover, macrophages accumulate within the lung during infection, resulting in phenotypic heterogeniety within AMs [6]. However, the AM phenotype marking the population permissive to *Coxiella* growth is not known.

Resident AM are characterized by high levels of CD11c surface integrin expression. This high level of integrin expression is dependent upon the lung microenvironment, as adoptive transfer of CD11c−/CD11b+ peritoneal macrophages into the lung of recipient mice, via intratracheal instillation, results in an upregulation of CD11c [7]. Similarly, monocytes recently recruited to the lung during inflammation initially express the typical monocytic lineage marker CD11b [8]. While AM from the naïve lung do not typically express observable levels of CD11b, certain stressors, such as hyperoxic conditions within the lung, can cause AM activation and subsequent upregulation of CD11b [8], [9]. Therefore, during infections, lung macrophage heterogeneniety can result from both an influx of recruited macrophages and changes in cell marker expression by non-elicited cells. Within this heterogeneous mix of resident and recruited cells, there exists a population of CD11c−/CD11b+ recruited cells, and a population of CD11c+/CD11b- resident cells, along with the newly observed subset of CD11c+/CD11b+ resident cells.

In addition to the high levels of CD11c expression on resident AM, these cells are remarkable for a high degree of autofluorescence when viewed with fluorescent microscopy or analyzed with fluorescence-activated cell sorting (FACS). This combination of CD11c expression and autofluorescence is recognized as characteristic of AM [10], [11], and allows for identification of resident AM within the naïve lung. However, upon infection, these characteristics do not provide discriminatory power sufficient to differentiate resident and recruited AM.

Discrimination of resident and recruited macrophages within the heterogeneous lung environment can be accomplished utilizing fluorescent lipophilic dye-labeling [12]. Briefly, the principle of this dye-labeling technique relies on the observation that AM turn-over rates within the murine lung vary from a half-life of one year under steady-state conditions to a half-life of two months during infection [13]. In contrast, the half-life of circulating murine monocytes has been reported to be twenty-two hours, and the average total time in circulation has been estimated to be forty-eight hours. [14], [15]. However, the amount of time monocytes remain in circulation is shortened under inflammatory conditions, with tissue recruited monocytes originating from the circulating population of monocytes [16]. Therefore, a dye-labeling strategy which introduces dye through intra-venous injection, labeling all cells present within the animal and then providing sufficient time for turn-over of circulating cells, will result in a selectively dye-labeled population of tissue resident cells.

In this study, we employed lipophilic dye-labeling to identify the importance of a resident population of macrophages within the lung in initial infection with *C. burnetii*. This population of AM was characterized by surface expression of both CD11c and CD11b and was comprised of long-term resident and newly resident macrophages responding to *C. burnetii* infection. This population was observable in a dose dependent fashion between six and forty-eight hours post-infection (PI) and was responsible for the majority of early cellular uptake of phase II Nine Mile *C. burnetii*. This subset of resident cells was also present in the context of phase I Nine Mile *C. burnetii* infection and the data presented conclusively supported a role for CD11c+ resident cells in the initiation and maintenance of phase I Nine Mile infection.

While recruited cells entering the lung subsequent to infection were capable of clearing phagocytosed bacteria, this was not true of resident AM. This cell-type not only was responsible for initial uptake of the bacteria, it was unable to clear bacteria during the subsequent infection. The result was a replicative niche for bacterial infection which influenced the overall course of infection. This was true irrespective of the phase variant responsible for infection and was not dependent on differential inflammatory responses induced by increasing doses of bacteria.

## Results

### Low Dose NMII Infection Resulted in Greater Increases in Numbers of Bacteria within the Lung, Compared to Inocula Numbers, Relative to High Dose Infection

**Figure 1:**
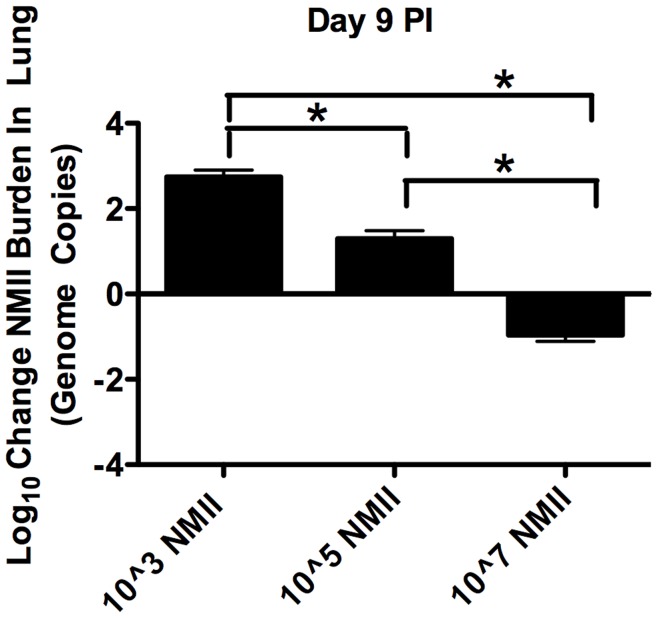
Lower doses of NMII resulted in greater relative bacterial numbers in lungs of C57BL/6 mice. NMII *Coxiella* bacterial burdens in lung tissue in groups of 4 C57Bl/6 mice infected with 1×10^3^ or 10^5^ or 10^7^ bacteria in the inocula were assessed, 9 days PI by quantitative RT-PCR. Data is expressed as Log10 change in total genome copies per lung. Significant increases in the final bacterial burdens were observed for both the 1×10^3^ and 10^5^ groups, while a significant decrease in the final bacterial burden was observed for the 1×10^7^ group (ANOVA p<0.05).

To assess whether resident AM could provide a niche exploited by bacteria during *C. burnetii* infection, quantitative RT-PCR was employed to determine growth rates of either 1×10^3^, 1×10^5^ or 1×10^7^ Nine Mile phase II *C. burnetii* (NMII) in lung tissue of groups of C57BL/6 mice 9 days PI. The results are shown as Log10 change in NMII burden (Log (Endpoint bacterial genome copies per lung/Inoculum)). Thus, the zero baseline represents the inoculum quantity for each respective group. As shown in Figure 1, when expressing pulmonary *Coxiella* burdens as the change in genome copies from the inocula number used to infect, mice infected with the lowest dose (1×10^3^) exhibited the greatest increase in *Coxiella* burdens at each time point. Mice given 1×10^5^ *Coxiella* exhibited less increase in *Coxiella* burden than those given the lowest dose but more than the mice given the highest dose. Mice given the highest dose exhibited an overall decrease in *Coxiella* burdens, relative to the inocula number. (ANOVA p<0.05) We obtained similar results in BALB/c mice at 2, 9, 16 or 24 days PI (Supplemental Figure S1).

These data were suggestive of a limited cellular niche present within the lungs that was susceptible to and permissive of infection. At higher infectious doses, bacteria appeared to completely fill this niche and bacteria that did not gain entrance to this niche were forced into cellular populations less conducive to survival and replication. The net result was reduced relative bacterial growth and increased bacterial clearance at higher initial inocula quantities.

It was possible that the results shown in Figure 1 could have been the result of high dose infections causing more bacterial dissemination from the lungs relative to low dose infections. However, we found that there were no significant differences in dissemination of NMII *Coxiella* from the lungs to the spleens of BALB/c mice, as indicated by spleen weights, at any of the infective doses used (Supplemental Figure S2). Indeed quantitative RT-PCR analysis of spleens indicated that no phase II *Coxiella* was present in the spleens of any of the mice (results not shown). In addition, there was little effect of dose on disease severity as indicated by similar body weight changes in the three groups of mice given the different doses of NMII (Supplemental Figure S2).

### NMII Infection Induced Cellular Recruitment and Phenotypic Change within AMs in a Dose and Time Dependent Manner

**Figure 2:**
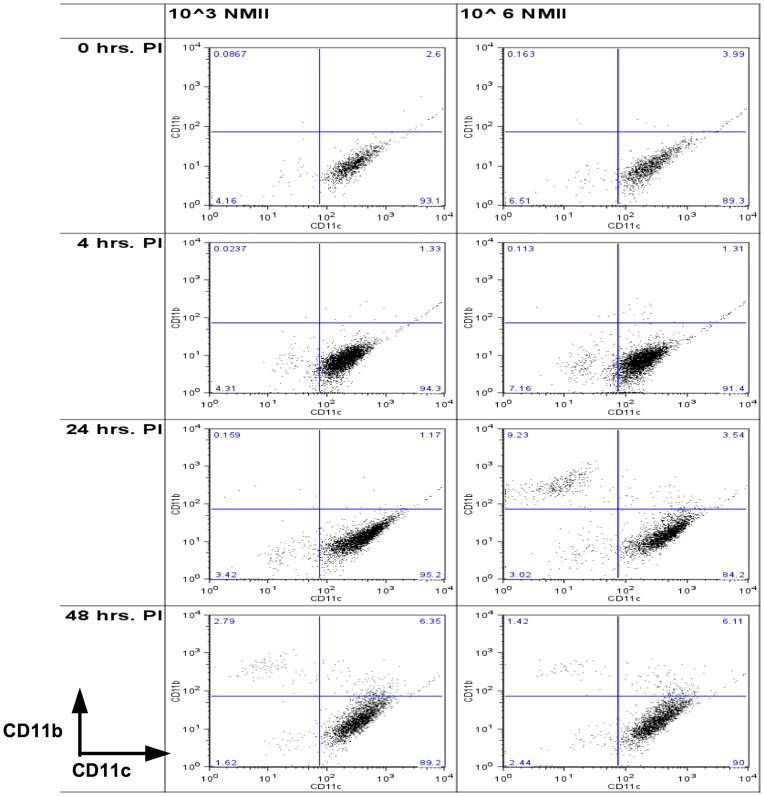
Pulmonary infection resulted in time and dose dependent alveolar macrophage phenotypic changes. Groups of 4 C57BL/6 mice were infected with either 10^3^ or 10^6^ NMII. Prior to infection, tissue resident AM in these mice were labeled with i.v. injection of the lipophilic dye DiD (see Figure 3 for further analysis). Mice were sacrificed 0, 4, 24 or 48 hrs PI. Resultant representative dot-plots of forward/side-scatter gated macrophages are shown. Uninfected mice exhibited a typical CD11b−/CD11c+ resident AM phenotype (top row of figure). Moving L-R in the second row of the figure, representative dot-plots are shown for mice infected 4 hours with 10^3^ or 10^6^ NMII respectively. Moving L-R in the third row of the figure, representative dot-plots are shown for mice infected 24 hours with 10^3^ or 10^6^ NMII. A CD11b+/CD11c- and a CD11b+/CD11c+ population of cells were present in the 10^6^ infection at this time. Moving L-R in the bottom row of the figure, representative dot-plots are shown for mice infected 48 hours with 10^3^ or 10^6^ NMII. Both infective doses exhibited a CD11b+/CD11c- and a CD11b+/CD11c+ population at this time.

To understand the kinetics of phenotypic changes of lung macrophages, the surface expression of both CD11c and CD11b integrins on AM (as defined by forward and side-scatter gating) was assessed by FACS on bronchoalveolar lavage fluid (BALF) cells at time-points directly following infection of groups of 4 C57BL/6 mice with low dose (1×10^3^) or high dose (1×10^6^ ) *C. burnetii*. For this experiment, the low infective dose was chosen to match the previous experiment (1×10^3^ NMII). The high infective dose was chosen to be 1×10^6^ NMII, because within the 48 hr. timeframe of the experiment, based on our results from the previous experiment, we felt that this dose would result in zero net change in bacterial burdens relative to the inocula quantity and thus provide the most appropriate comparison dose. Tissue resident AM, prior to infection (0 hrs), exhibited high levels of CD11c expression (Figure 2), and these high levels of expression were observed at all time-points and doses subsequent to infection on two AM subsets, the CD11b−/CD11c+ and CD11b+/CD11c+ AM. At the low dose (10^3^), by forty-eight hours, CD11b expression was upregulated on the CD11c+ population, and CD11b+/CD11c- macrophages had accumulated. At the higher dose (10^6^), already by twenty-four hours, CD11b expression was upregulated on the CD11c+ population, and CD11b+/CD11c- macrophages had accumulated. Thus, there was a dose and time dependent appearance of both a CD11b+/CD11c+ and a CD11b+/CD11c- subset of cells within the overall AM population leading to the observed heterogeneity of the AM population during infection.

### During NMII Infection, CD11b−/CD11c+ and CD11b+/CD11c+ AM Populations were Predominantly Comprised of Cells Resident Prior to Infection, while CD11b+/CD11c- macrophages were Cells Recruited after the Onset of Infection

The time-course of macrophage phenotypic response to pulmonary *C. burnetii* infection, presented in Figure 2, suggested that the CD11b+/CD11c+ macrophage population seen after infection was the resident population, and the CD11b+/CD11c- macrophage population was comprised of newly recruited monocytes. To determine the residency or recruitment origins of lung macrophages in the context of infection, mice in the above kinetics experiment were also given intravenous (i.v.) injection of DiD lipophilic dye prior to infection. This lipophilic dye-labeling served to distinguish tissue resident AM from newly recruited macrophages entering the lung during infection. In this way, we were able to both verify AM origin and quantify heterogeneity within the AM subsets phenotypically defined in Figure 2.

**Figure 3:**
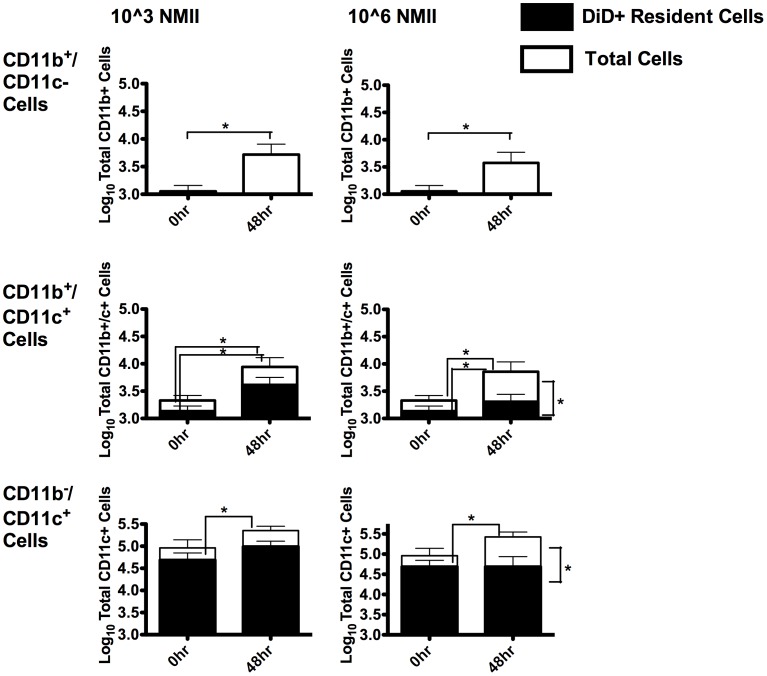
Resident AM predominantly expressed CD11c, an AM subset co-expressed CD11b during NMII infection. The BALF samples collected and presented in Figure 2 were further analyzed for the presence of the lipophilic dye DiD. Gated macrophages in the FACS analysis were divided into dye positive and dye negative samples by comparison with an infected unstained control (data not shown). The resultant populations were then expressed as total cell numbers. The results indicated that, AM heterogeneity within the lung, during infection, was characterized by the presence of three main phenotypic AM subsets. Across both the 10^3^ and 10^6^ infective doses, a significant increase in CD11b+ cells was observed between 0 hrs and 48 hrs PI (p<0.05 ANOVA). These cells were newly recruited to the lung, following the onset of infection. There was a heterogeneous population of resident and recruited cells expressing a CD11b+/CD11c+ phenotype. Finally, there was a resident population of CD11c+ cells that were present within the lung prior to infection. In either infective dose, there was a significant increase in total cells at 48 hrs PI (p<0.05 ANOVA).

Macrophages present within the lung during infection were comprised of three main phenotypic subsets, characterized by CD11b and CD11c expression (Figure 3). Detectable numbers of CD11b+/CD11c- cells had accumulated in the lungs of infected mice by 48 hours, irrespective of dose. None of the CD11b+/CD11c- lung macrophages, regardless of time of infection or dose, were DiD positive. Thus, these macrophages were not resident within the lung at the onset of infection. Low numbers of CD11b+/CD11c+ macrophages were present in the lung before infection; however, this population increased by 48 hours at both infective doses. Regardless of time or dose, approximately half of this cell population was comprised of DiD+ cells resident within the lung prior to infection. The combined CD11b+/CD11c+ and CD11b−/CD11c+ populations of lung macrophages in low dose infection remained relatively constant in numbers and consisted primarily of DiD+ cells resident prior to infection. Thus, in the context of low dose infection, the CD11b+/CD11c+ cell population is predominantly comprised of resident cells that have upregulated CD11b. In contrast, infection with 1×10^6^ NMII resulted in a significant increase in non-resident CD11b+/CD11c+ cells, relative to resident CD11b+/CD11c+ cells, at 48 hrs. PI. These results are consistent with a larger inflammatory response in the context of higher infective doses.

These results highlighted the upregulation of CD11b in a subset of tissue resident AM responding to infection. Further, these results highlighted a dose dependent recruitment of CD11b cells entering the lung in response to infection. The net result of this recruitment and phenotypic response to infection was a heterogeneous population of CD11b+/CD11c+ cells present in the alveolus during *C. burnetii* infection.

### CD11c+ Resident Cells were Responsible for NMII Coxiella Uptake and Persistence

**Figure 4:**
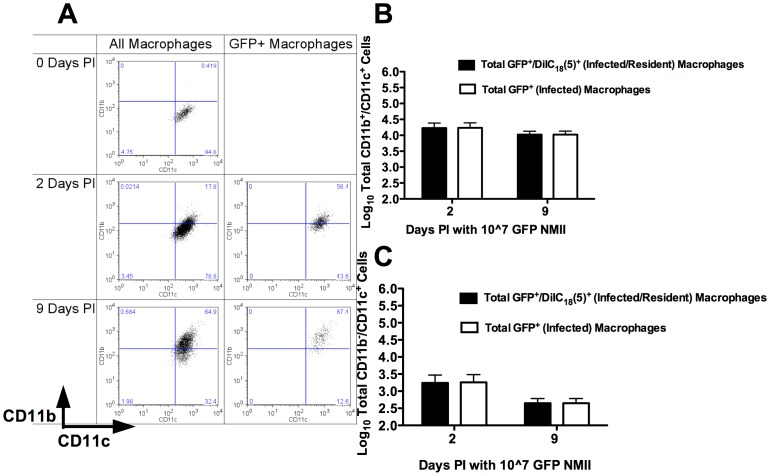
CD11b+/CD11c+ AM were the most highly infected cell-type two and nine days post-infection with NMII. Groups of 5 C57BL/6 mice were infected with 1×10^7^ GFP expressing NMII and sacrificed either 2 or 9 days PI. BALF was collected and FACS was performed on the samples. Representative plots gated for AM on forward/side scatter are shown from groups of 5 mice. A) Beginning at 2 days and peaking by 9 days PI, a significant CD11b+/CD11c+ AM population was observed in NMII infected animals. It was this population, which predominated as the infected macrophage population. B) The majority of macrophages were CD11b+/CD11c+ cells. Of these CD11b+/CD11c+ cells, nearly all were infected cells resident within the lung prior to infection (as determined by DiIC_18_(5) lipophilic dye staining and GFP fluorescence). There was a minor population of resident CD11b−/CD11c+ cells that was also infected 2 days PI. This population was not observable 9 days PI.

To determine whether NMII infection of AMs occurred within the resident or recruited AM population, groups of 5 C57BL/6 mice i.v. injected with DiIC_18_(5), to label tissue resident cells, were infected with 1×10^7^ GFP NMII (this dose was chosen because, in our hands, GFP expressing *C. burnetii* was slightly less infectious than wild-type *C. burnetii*). At two and nine days PI, BALF cells were subjected to FACS analysis with macrophages identified based on forward and side-scatter characteristics, as well as SiglecF staining [17], [18] (Figure 4- SiglecF gating not shown). As shown in Figure 4A (left-hand column), with respect to the entire AM population, there was a marked increase in the CD11b+/CD11c+ population from 0 days PI to 9 days PI. This increase was observable as early as 2 days PI, and by 9 days PI the CD11b+/CD11c+ population predominated in BALF. This increase in the GFP+/CD11b+/CD11c+ population was accompanied by a concomitant decrease in the size of the CD11b−/CD11c+ population across the same time-course.

When the phenotype of only GFP+ macrophages was assessed, marking those cells having taken up *C. burnetii*, a similar increase in the CD11b+/CD11c+ population across the time-course of infection was observed (Figure 4A- right-hand column). At 2 days PI, both CD11b+/CD11c+ and CD11b−/CD11c+ macrophages were positive for the presence of *C. burnetii*. By 9 days PI, GFP+ macrophages were predominantly CD11b+/CD11c+.

At 2 and 9 days PI, nearly all of the GFP+/CD11b+/CD11c+ and GFP+/CD11b−/CD11c+ macrophages were also DiIC_18_(5)+, indicating that they were tissue resident cells prior to the onset of infection (Figure 4B and Figure 4C). However, by 9 days PI, GFP+ macrophages predominated in the CD11b+/CD11c+ DiIC_18_(5)+ resident macrophage population (Figure 4B). This is consistent with the above-mentioned phenotypic change (Figure 4A) during infection whereby resident AM upregulate CD11b during infection.

These results indicated that the resident AM was both susceptible to and permissive of NMII infection. Further, within the resident AM population, the subset of resident AMs, which underwent a characteristic phenotypic change defined by increased CD11b expression, were the resident AM most highly susceptible to *C. burnetii* infection. Alternatively, the AM population most susceptible to infection upregulated CD11b expression.

### Increased Pulmonary Inflammation was Insufficient to Account for the Observed Deficiency in Bacterial Replication during High Dose Coxiella Infection

**Figure 5:**
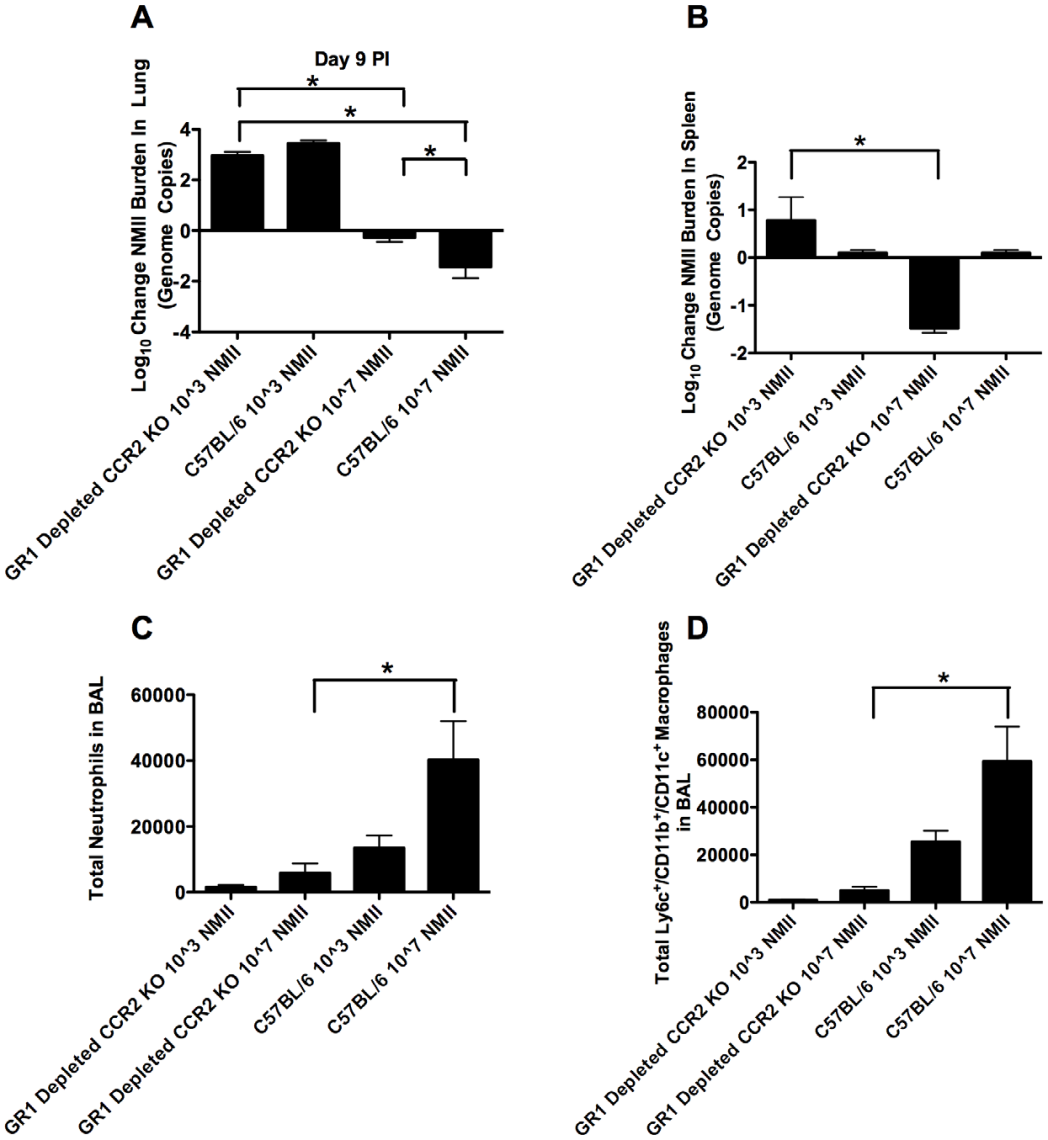
Inflammation is not sufficient to account for decreased relative bacterial numbers in higher dose infection. A) C57BL/6 mice or CCR2 KO mice receiving Gr1-depleting antibody were infected with either 10^3^ or 10^7^ NMII *Coxiella*. Bacterial burdens in lung tissue were assessed 9 days PI by quantitative RT-PCR. Data is expressed as Log10 change in total genome copies per lung. Groups were comprised of 5 mice with Mean and SEM plotted for each group. Low dose infection of both wild-type mice and KO mice receiving depleting antibody treatment resulted in greater bacterial replication relative to either wild-type mice or KO mice receiving depleting antibody treatment and high dose infection. B) Either low or high dose infection of KO mice receiving depleting antibody treatment exhibited measurable bacterial burdens in spleen tissue 9 days PI; whereas, wild-type mice at either infectious dose did not exhibit bacterial burdens in the spleen at the same time-point. C) Gr1-depletion resulted in a significant reduction in the number of neutrophils recruited to the lung at day 9 PI in high dose infection. D) CCR2 KO animals exhibited a significant decrease in the number of recruited Ly6c+/CD11b+/CD11c+ monocytes at day 9 PI in high dose infection. (p<0.05 ANOVA).

To address the possibility that high dose infection was altering the immune status of the resident AM and therefore resulting in the observed reduction in bacterial replication compared to low dose infection, an experiment to block inflammatory cellular influx into the lung during high dose infection was performed. Groups of 5 C57BL/6 mice or CCR2 KO mice having received Gr1-depleting antibody were infected with either 10^3^or 10^7^ NMII *Coxiella*. The CCR2 KO mice that received Gr1-depleting antibody were anticipated to lack the ability to recruit either inflammatory monocytes or neutrophils to the site of infection. At day 9 PI, bacterial burdens in lung tissue were assessed by quantitative RT-PCR. As we found previously (Figure 1), in the WT mice not depleted of Gr-1+ cells, the low dose NMII resulted in more bacterial proliferation relative to the inocula given than did high dose infection. In the Gr-1+ cell-depleted CCR2 KO mice, the results were similar, with the low dose resulting in relatively more growth of NMII, compared to the high dose. In low dose infection, no significant difference in the change in bacterial burdens was observed between wild-type and Gr-1+ cell-depleted CCR2 KO mice. Both groups exhibited greater than a 2-log increase in bacterial burdens over the inoculum number (Figure 5A). This similarity in results, regardless of blockade of inflammatory cell influx, could be due to the minimal overall influx of inflammatory cells during low dose infection (Figure 5C and Figure 5D). In the high dose infection, there was a significant decrease in both recruited neutrophils (Figure 5C) and recruited monocytes (Figure 5D) in the Gr-1+ cell-depleted CCR2 KO mice. This blockade of inflammatory cell influx did result in less of an overall reduction in bacterial burdens from the inoculum number in the Gr-1+ cell-depleted CCR2 KO mice, compared to wild-type mice. Importantly, any increase in bacterial replication due to the blockade of inflammatory cell influx was insufficient to increase final bacterial burdens in these animals beyond the initial inoculum number. Thus, differences in inflammation between high dose and low dose infection could not account for all of the differences in growth of *Coxiella* observed between the low and high dose infections. In addition, the results indicate that in the mouse, the influx of new macrophages into the lungs does not supply a source of cells susceptible to NMII infection, further supporting that resident AM are the susceptible macrophage phenotype (Figure 5A). Both low and high dose infection of KO mice receiving depleting antibody treatment resulted in measurable bacterial burdens in spleen tissue 9 days PI; whereas, wild-type mice at either infectious dose did not exhibit bacterial burdens in the spleen at the same time-point (Figure 5B). This indicates that inflammation plays a critical role in limiting the dissemination of NMII from the lungs to the spleen. Gr1-depletion did indeed result in a significant reduction in the number of neutrophils recruited to the lung at day 9 PI in high dose infection (Figure 5C). Likewise, CCR2 KO animals did exhibit a significant decrease in the number of recruited Ly6c+/CD11b+/CD11c+ monocytes at day 9 PI in high dose infection (Figure 5D).

Taken together, these data are consistent with the resident AM providing a limited niche for both the establishment and maintenance of NMII infection. These data are not consistent with the idea that alteration of resident AM function resulting from inflammation in high dose infection is sufficient to account for the entire observed reduction in bacterial replication in the context of high dose infection. Further, these data suggest that recruited inflammatory cells and particularly inflammatory monocytes did not act as a pool of cells in the lungs susceptible to NMII infection, but in fact these cells played a minor role in the control of NMII infection. This control could be due either to the ability of inflammatory monocytes to directly clear bacteria present in the lung, or as a result of inflammatory monocytes blocking bacterial dissemination.

### Low Dose NMI Infection Resulted in Greater Increases, Compared to Inocula Numbers, in Bacteria Numbers within the Lung Relative to High Dose Infection

**Figure 6:**
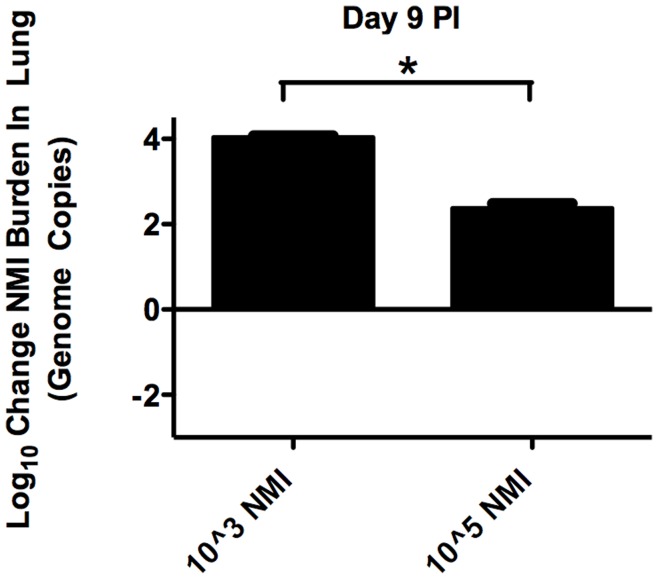
Lower doses of NMI resulted in greater relative bacterial numbers in lungs of C57BL/6 mice. NMI *Coxiella* bacterial burdens were assessed 9 days PI in groups of 5 C57BL/6 mice infected with either 1×10^3^ or 10^5^ starting inocula. Bacterial burdens in lung tissue were assessed by quantitative RT-PCR. Data is expressed as Log10 change in total genome copies per lung. A significant increase in the final bacterial burden was observed in the lower starting inocula group for this mouse strain (Student T test p<0.0001).

To assess whether resident AM could provide a niche exploited by bacteria during Nine Mile phase I *C. burnetii* (NMI) infection, similar to what we observed for Nine Mile phase II *C. burnetii,* quantitative RT-PCR was employed to determine growth rates of either 1×10^3^ or 1×10^5^ NMI in lung tissue of groups of C57/BL6 mice at 9 days PI. As shown in Figure 6, when expressing pulmonary *Coxiella* burdens as the change in genome copies from the inocula number used to infect, mice infected with the lowest dose (1×10^3^) exhibited a greater increase in *Coxiella* burdens relative to mice given the higher dose (1×10^5^) (ANOVA p<0.05). We obtained similar results in BALB/c mice at 2, 9, 16, or 23 days PI (Supplemental Figure S3).

Interestingly, with NMI infection we were able to isolate bacterial DNA from the spleens of infected BALB/c mice. At day 9 PI, there was a significant increase in splenic bacterial burden for the 1×10^3^ NMI group, relative to the other groups. The changes in bacterial burdens within the spleen, for all groups, appeared to follow a general trend of lagging changes in bacterial burden observed in the lung. This is quite consistent with the idea of pulmonary replication within a limited cellular niche followed by breakout from macrophages and dissemination of the bacteria. In particular, bacterial burdens within the lung exhibited an increase from day 9 to day 16 PI for the mid dose infection (Supplemental Figure S3). At day 23 PI, bacterial burdens in the spleen exhibit an increase, relative to day 16 PI, for the mid dose infection (Supplemental Figure S4). Again, as with NMII infection, these data indicated that high infectious doses exhibited decreased replicative efficiency relative to low infectious doses. This difference could not be solely accounted for by an increased dissemination from the lung of NMI organisms after high dose infections. However, there does appear to be increased bacterial dissemination at all infective doses, and notably at higher doses, in NMI infection compared to NMII infection. In fact, this may be a key factor contributing to the observed differences in virulence for the two bacterial phases.

### Phenotypic Responses of AM to NMI Infection were Similar to those of AM to NMII Infection, with CD11b+/CD11c+ and CD11b−/CD11c+ AM Populations Predominantly Comprised of Resident Cells

Having determined both the overall phenotypic profile during infection and the phenotypic profile associated with susceptibility to NMII infection, we determined whether the observed AM phenotypic response was consistent across *C. burnetii* phase variants.

**Figure 7:**
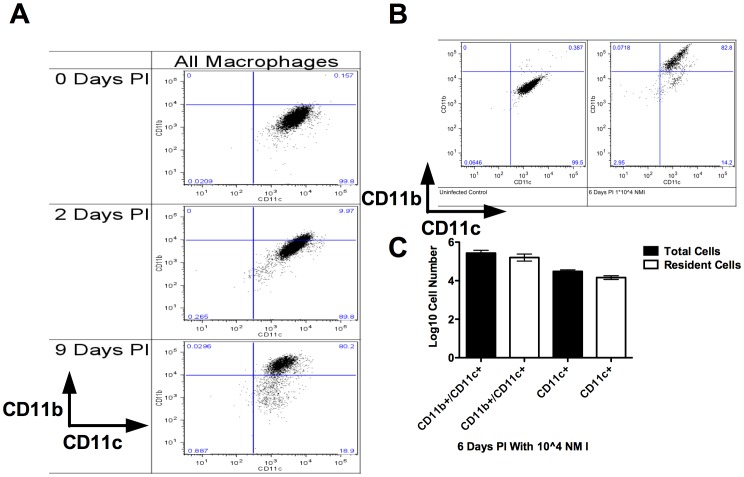
Resident AM predominantly expressed CD11c, an AM subset co-expressed CD11b during NMI infection. A) Two days PI there were CD11b−/CD11c+ and CD11b+/CD11c+ AM subsets present within the groups of 5 NMI infected C57BL/6 mice. By 9 days PI, the CD11b+/CD11c+ AM subset predominated as the major AM subset present within the NMI infected animals. B) By 6 days PI, the CD11b+/CD11c+ AM subset was already predominating as the major AM subset present within the NMI infected animals. C) In NMI infection, both the CD11b+/CD11c+ and CD11b−/CD11c+ macrophage populations were predominantly comprised of cells resident within the lung prior to infection (as determined by DiD lipophilic dye staining). This was analogous to the phenotypic profile observed in NMII infection.

Due to the limited capacity for FACS analysis in our BSL-3 facility, and because a GFP expressing phase I *C. burnetii* was not available, we were not able to directly determine the phenotype of AM infected with NMI by FACS. Thus, we compared the AM phenotypic response in NMII infected mice to that in NMI infected mice. To determine whether the phenotypic responses of AM to *C. burnetii* infection were consistent across phase variants, groups of 5 C57BL/6 mice were infected with low dose (1×10^4^– chosen to be intermediate between the two doses causing bacterial replication in the previous experiment and still sufficient to cause an inflammatory response observable by FACS) NMI. Phenotypic changes in CD11b expression during NMI infection were analogous to those observed during the course of NMII infection (Compare Figure 4A and 7A).

As shown in Figure 7A, two days PI, there was a marked increase in the CD11b+/CD11c+ population of cells, defined as macrophages based on SiglecF expression (parental gating strategy not shown), relative to the naïve control. By nine days PI, this double positive cell-type predominated in the BALF. The progression of phenotypic change from 2 to 9 days PI mirrors the increase in bacterial replication shown across the same timecourse (Supplemental Figure S3). This further supports the claim that it is specifically the CD11b+/CD11c+ AM population that is both most susceptible to and permissive of infection.

In a separate experiment, groups of 5 DiD lipophilic dye-labeled C57BL/6 mice were infected with 1×10^4^ NMI and killed at 6 days PI. The CD11b and CD11c staining of the AM (Figure 7B) was similar to the staining observed on AM from mice killed at day 9 PI (Figure 7A). Further, this CD11b+/CD11c+ AM population is comprised almost exclusively of tissue resident AM (as assessed by DiD staining and presented in Figure 7C).

These results indicated that, with respect to AM phenotypic responses, the two *C. burnetii* phase variants elicited similar immune responses. This was consistent with the previously mentioned speculation that both bacterial phase variants present similarly to the host immune system, and that there is an intrinsic difference in down-stream host immune response that is responsible for the observed differences in virulence. More importantly, this also suggested that, analogous to our findings in NMII infection, the resident AM might provide a required niche for both NMII and NMI infection.

### NMI Coxiella Infected Cells were Phenotypically Consistent with Resident AM

**Figure 8:**
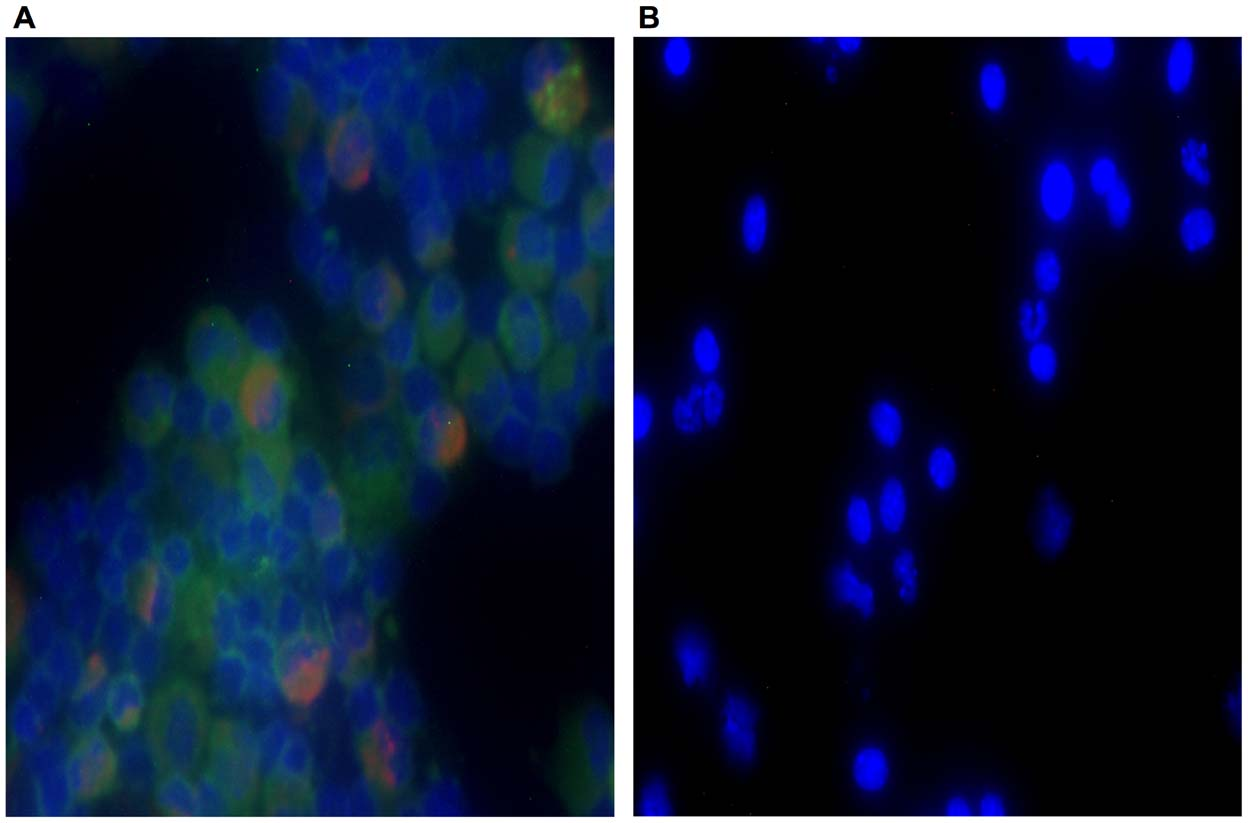
NMI infected cells expressed CD11c+ and were consistent with a resident AM phenotype. Groups of 5–6 C57BL/6 mice were infected with 1×10^4^ NMI. Twelve days PI, BALF fluid was obtained and histology of cytospin preparations was performed. A) Twelve days PI with 1×10^4^ NMI, infected cells were identified by the presence of *Coxiella* (stained red) and CD11c expression (stained green). Results were consistent with infected cells being a resident AM phenotype. B) Twelve days PI with 1×10^4^ NMI, BALF cells were adhered to slides and were not stained as a control for autofluorescence. Representative photomicrographs are shown.

Given the similarities in the characteristic changes in phenotypic expression of AM subsets throughout the course of NMII and NMI infection, we hypothesized that during NMI infection the resident AM would be the cellular subset susceptible to infection. To test this, we infected groups of 5–6 C57BL/6 mice with 1×10^4^ NMI (dose chosen to match the previous experiment) for 12 days. At 12 days PI, cytospin slides of BALF cells from the two groups of mice were prepared and AM were stained for both *C. burnetii* and CD11c expression (Figure 8A-representative sample, Figure 8B-negative control).

NMI infected AM at day twelve PI were characterized by CD11c staining. That the infected AM expressed high levels of CD11c, was suggestive of lung residency prior to infection.

## Discussion

Our results indicated that a subset of tissue resident AM was both susceptible to and permissive of *C. burnetii* infection. This population of cells underwent a characteristic phenotypic upregulation of CD11b expression subsequent to infection. More importantly, this population represented a limited cellular niche pivotal in the initiation and maintainence of infection. The limited nature of this population may also account for the general limited susceptibility of mice to *C. burnetii* infection.

*C. burnetii* has long been recognized as highly infectious, with literature reports suggesting that as few as one organism may be capable of causing infection in humans [19], [20]. Moreover, a recent study has shown a strong statistical correlation between bacterial infectivity and the ability to survive intracellularly in immune cells. This study suggested that bacteria with high ID50’s overwhelm the immune system through rapid growth, while bacteria with low ID50’s evade the immune response [21]. Such an evasion strategy would be very consistent with the characteristics of *C. burnetii* infection and would be consistent with the idea of a limited cellular niche susceptible to and permissive of infection.

Guinea pigs are often used as a model system to study *C. burnetii* infection, as these animals manifest dose dependent symptoms which closely recapitualte disease in humans [22]. However, SCID mice also exhibit enhanced susceptibility to both phase I and phase II *C. burnetii* infection and have been used as a susceptible mouse model of disease [23]. Within animal models of disease, bacterial strain differences affecting susceptibility to infection have been noted [24]. Specifically, while SCID mice appeared susceptible to all *C. burnetii* isolates tested in the study, various groups of isolates exhibited differing levels of infectivity in guinea pigs. In particular, Nine Mile isolates (the isolate used for all studies presented here-in) proved to be infectious regardless of the animal model employed. Moreover, while no wild-type mouse strain is particularily susceptible to phase II *C. burnetii* infection, mouse strain differences have been noted to play a role in overall susceptibility. For example, C57BL/6 mice were shown to be less susceptibile to NMII infection than BALB/c mice [25]. In light of our results in this study, these differences in susceptibility could be consistent with a difference in the ability to control bacterial break-out from the susceptible AM niche. Further, this would be consistent with previous work performed in our lab showing the requirement for adaptive immunity in the control of *C. burnetii* infection [26]. Thus, while the permissive and susceptible AM population plays a central role in influencing the overall course of infection, the ultimate outcome of infection is determined by the adaptive immune response. The interaction between the early susceptible innate AM population and the later adaptive response is an area for on-going research.

We observed that low doses of NMII were not only cleared less efficiently in early infection, but also appeared to replicate more efficiently, relative to high dose infections (Figure 1 and Supplemental Figure S1). This suggested that a cellular niche within the lung was acting to harbor *C. burnetii*, providing an environment conducive to bacterial replication. At low doses most of the bacteria were able to exploit this niche; at high doses some of the bacteria were probably forced into less favorable niches. In deriving this conclusion, it is important to note that we were unable to recover bacteria from the spleens of animals infected with any dose of NMII across any of the timecourse of infection (see Supplemental Figure S2 for surrogate measures of systemic infection). This was a strong indication that our observations of dose dependent differences in bacterial clearance and replication within the lung could not be merely attributed to a greater rate of bacterial dissemination in high dose infected animals. When considering the data from the onset to conclusion of infection, we did not observe any dose-related systemic differences in infection. Instead, we observed a lung localized difference in bacterial burden where low dose innocula led to increased bacterial burdens relative to the infective dose used. Similar results were obtained in the context of NMI infection, with the notable exception of increased bacterial dissemination across infective doses (data not shown). This is consistent with the increased virulence of the NMI phase variant.

The role which the micro-environment of the lung plays in immuno-regulation of the AM populations, and by extension, the effect it has on the susceptibility of these populations, is becoming increasingly recognized. For example, immunomodulatory interactions of CD200 expressed on airway epithelium, and CD200R expressed on alveolar myeloid cells including AM, downregulates alveolar myeloid cell and AM function [27], [28], whereas inflammatory monocytes accumulating in the lungs would not come under this control. Our data suggested that *C. burnetii* may take advantage of the suppressed state of resident AM, preferentially infecting this cell population.

As mentioned, we had reason to believe that *C. burnetii* was exploiting a cellular niche in order to survive, and this niche was likely to be found within the AM population. In this regard, we observed a phenotypic switch in the infected macrophage population such that infected CD11c+ cells began to upregulate CD11b surface integrin expression (Figure 2). This observation was made during FACS analysis of cells from lavage fluid. This analysis provided a distinguishing phenotypic description of infected cells. However, this analysis alone provided limited data regarding whether the infected cells were tissue resident within the lung prior to the onset of infection. Therefore, this approach was insufficient to identify the putative cellular niche susceptible to bacterial infection within the heterogeneous cellular environment of the lung. Still, the approach was sufficient to provide direction to future analysis, specifically suggesting that the resident AM could account for a susceptible cellular niche, consistent with our hypothesis.

Macrophage heterogeneity within the alveolus has been recognized for some time. This heterogeneity includes a mixture of CD11b−/CD11c+ expressing resident cells and CD11b+/CD11c- expressing recruited cells. Additionally, although literature directly addressing phenotypic changes of lung macrophages during infection is limited [29], we show here that a population of CD11b+/CD11c+ cells comprised of both previously CD11b−/CD11c+ long-term resident and comparatively newly resident previously CD11b+/CD11c- cells exists within the alveolus of the lung directly following *C. burnetii* infection (Figure 3).

To determine whether infected AM were resident in the lungs at the time of infection, resident AM subsets were identified, utilizing a dye-labeling technique. Davidson et. al. found that when introducing the fluorescent lipophilic dye DiI systemically through tail-vein injection, tissue resident macrophages were dye bright in recipient mice six days after injection, and circulating monocytes were dye negative to dye low [12]. Utilizing this method, we were able to discern both resident and recruited macrophage populations within the lung. Then, by employing a GFP expressing NMII bacteria, we were additionally able to identify members of these various phenotypic subsets as either *C. burnetii* infected or non-infected (Figure 4). Our findings show that the CD11b+/CD11c+ resident AM population observed during infection was the most highly susceptible population. While it is possible that CD11c+ cells harboring fewer bacteria were indistinguishable from the autofluorescent background, the highly infected CD11b+/CD11c+ population of cells was clearly identifiable.

These findings are consistent with the work of Garn et. al., who found that after NO_2_ exposure CD11b+ cells predominate in the lung macrophage population [30]. Here, we not only show an influx of CD11b+/CD11c- cells upon induction of infection, we show that these recruited cells upregulate CD11c upon entry into the lung during the transition to resident AM, thus providing an *in vivo* finding within the context of pulmonary infection to corroborate the adoptive transfer work performed by Guth et. al., that concluded CD11c- monocytes upregulate CD11c upon migration into the lung alveoli [7]. Moreover, and most importantly, we show an upregulation of CD11b by a subset of resident AM subsequent to infection. It is this subset of resident cells which predominates in the early uptake of NMII *C. burnetii* and becomes functionally relevant in the establishment of infection.

Because high dose infection most certainly will result in increased pulmonary inflammation over that induced by low dose infection, we devised experiments to limit inflammation in the context of high dose infection. In this way we could investigate whether the deficiencies in bacterial replication observed during high dose infection were the result of inflammation limiting bacterial growth, as opposed to a saturation of the permissive niche by high dose infection. In our model, we took advantage of CCR2 knock-out mice lacking a functional receptor for monocyte chemoattractant protein-1 (MCP-1). These mice have been shown to be difficent in both monocyte recruitment [31] and clearance of *Listeria monocytogenes*, an intracellular bacterium [32]. While some diminishment of recruited inflammatory monocytes has been observed with Gr1-depletion [33], we expected this to be minimal. Therefore, Gr1-depletion alone was insufficient to inhibit monocyte recruitment into the lungs upon high dose infection. However, by employing Gr1-depletion in CCR2 KO animals we were able to significantly reduce both neutrophil recruitment and Ly6c+/CD11b+/CD11c+ monocyte recruitment (Figure 5) while leaving the resident AM population unaltered. The Ly6c+/CD11b+/CD11c+ cells have been variously described as inflammatory monocytes, inflammatory dendritic cell precursors, or inflammatory dendritic cells [34], [35], [36]. Blockade of inflammatory cell influx to the lung did result in significantly less clearance (or enhanced growth) of high dose NMII. However, at the low dose, the absence of inflammation had no significant effect. Overall, the absence of inflammation did not result in a relative increase in growth in the high dose sufficient to effect NMII burdens to the extent observed in the mice receiving low dose infection. Thus, differences in inflammation between high dose and low dose mice could not account for all of the differences in *Coxiella* growth observed between the low and high dose infections. Further, the influx of new macrophages into the lungs did not appear to supply a source of cells susceptible to NMII infection. This observation is consistent with resident AM providing a limited susceptible and permissive niche during infection. This niche appeared to be unchanged in a pro-inflammatory environment. Moreover, these results indicated that the recruited monocyte population played an important role in the control of infection through facilitating bacterial clearance and possibly altering bacterial dissemination from the lung to the spleen.

We recapitulated our NMII findings in the context of NMI infection and showed that low dose infection leads to enhanced bacterial inocula replication within the lung (Figure 6). Unlike NMII infection in wild-type animals, but similar to NMII infection in CCR2 KO Gr1-depleted animals, NMI infection resulted in bacterial dissemination to the spleen at all infective doses (Supplemental Figure S4). In fact, this difference in bacterial dissemination could account for the observed differences in virulence between NMII and NMI.

Due to the technical constraints of working with NMI in BSL-3 conditions, we could not directly show that it it is the CD11b+/CD11c+ resident AM which is the key cellular niche for phase I infection. However, we were able to conclusively show that it is a CD11c+ resident AM which harbors NMI bacteria (Figure 8). The phenotypic profiling during the time-course of NMI infection is consistent with that of NMII infection, further implicating the CD11b+/CD11c+ population of resident cells as the permissive niche (Figure 7).

Both phase II and phase I bacteria exhibited enhanced growth and persistence when infective doses were lower, as seen in the dose response experiments. Importantly, mice did not succomb to infection of either phase at any of the doses used. We observed that the phenotypic response of the AM were similar in both NMII and NMI infection. Moreover, most of the infected AM in NMII infection were cells resident within the lung prior to infection. Likewise, most of the NMI infected cells expresed CD11c and were phenotypically consistent with being resident AM. Furthermore, the dose dependent growth of Coxiella could not be attributed solely to differences in either inflammation or dissemination. Taken together, this data is highly suggestive that the cellular niche susceptible to and permissive of both phase II and phase I infection is a subset of the resident AM population.

In conclusion, the resident AM provides a pivotal niche involved in maintaining *C. burnetii* infection. Within this resident AM population a subset of cells, characterized by surface expression of both CD11c and CD11b integrins, was primarily responsible for the initial uptake of *C. burnetii*. The role of the resident AM as a susceptible and permissive niche for *C. burnetii* infection was conserved across bacterial phase I and phase II variants. Although *C. burnetii* infection causes an influx of inflammatory macrophages into the lungs of mice, this population was not permissive to *C. burnetii* growth.

## Materials and Methods

### Ethics Statement

All experimental procedures performed were in accordance with Guide for the Care and Use of Laboratory Animals of the National Institutes of Health and approved by the Institutional Animal Care and Use Committee at Montana State University, Bozeman, MT (Protocol permit # 2011–12). All efforts were taken to minimize pain and suffering.

### Bacterial Strains

*Coxiella burnetii* Nine Mile phase I (NMI strain RSA493), Nine Mile phase II (NMII strain RSA439) and Nine Mile phase II GFP (NMII GFP phase II, clone 4 strain RSA439/Tn7-CAT-GFP) were kindly donated by Robert Heinzen (Rocky Mountain Labs NIH/NIAID, Hamilton, MT).

### Animals

Mice used were male mice between 6 and 8 weeks of age and obtained from NCI, Rockville MD or in-house breeding colonies established at Montana State University with founder mice obtained from Jackson Laboratory, Bar Harbor ME. Dose response experiments (Supplemental Figures S1, S2, S3, and S4) were performed in BALB/c mice; all other experiments were performed in C57BL/6 mice. Experiments with CCR2 KO mice were performed using mice from an in-house breeding colony established at Montana State University from commercially available founder B6.129S4-Ccr2tm1Ifc/J mice (stock#004999) obtained from Jackson Laboratory, Bar Harbor ME.

### Intra-tracheal Infection of Mice

For all experiments, mice were lightly anaesthetized with isoflurane and inoculated intratracheally (i.t.) with final concentrations of NMI or NMII genome copies suspended in 100 µl sterile phosphate-buffered saline (PBS).

### Collection of Bronchoalveolar Lavage

BALF was collected as previously described [26]. Briefly, mice were given an intraperitoneal (i.p.) injection of a Phenobarbital solution and exsanguinated. Following this, mice were cannulated via a small tracheal incision and the lungs were flushed with aliquots of Hanks’ balanced salt solution with 3 mM/liter EDTA in a final volume of 5 ml.

### Determination of Bacterial Burden by Quantitative PCR

To assess and compare bacterial burdens in lung tissue under the various experimental conditions, quantitative PCR was performed as previously reported [26], [37]. Briefly, whole sample DNA was extracted from lung or spleen tissue with a Qiagen DNeasy blood and tissue kit (Qiagen Inc USA, Valencia, CA) following manufacturer’s protocols. Bacterial DNA was amplified in a quantitative real-time PCR reaction with SYBR green PCR master mixture (Applied Biosystems, Foster City, CA). Genome copies were determined by amplification of *rpoS* gene copies as previously reported [26], [37]. Reactions were performed with an Applied Biosystems 7500 real-time PCR system (Applied Biosystems).

### Calculation of Body Weight and Spleen as a Percent of Body Weight

Mice were weighed daily on a Denver Instrument Company WE Series 400 digital scale (Denver Instrument Bohemia, NY). Body weights were recorded in grams and % change in body weight was calculated by the following formula: (Final Body Weight (g)/Initial Body Weight (g)) * 100. At each endpoint, spleens were removed and weighed. The resultant weight was recorded in grams and divided by total body weight. This final number was multiplied by 100 and reported as Spleen as a % of body weight.

### Gr1+ Cell Depletion

Gr1+ cell depletion was performed by daily i.p. injection with 250 µg RB6-8C5 hybridoma derived GR1-depleting antibody produced in the Harmsen laboratory (described previously by Dr. R. Coffman of DNAX Research Inst. Palo Alto, CA [38], [39]) and suspended in 500 µl sterile PBS.

### Lipophilic Dye-labeling of Resident Cells

Mice were labeled with lipophilic dye via i.v. injection of DiD or DiIC_18_(5) Vybrant Cell-labeling dye (Molecular Probes, Eugene OR) suspended at a 10 fold dilution from stock in a 2% BSA carrier to a final volume of 100 µl per injection. Prior to any subsequent experimental procedures, mice were rested for one week to ensure that cells newly recruited to the site of infection would be dye low to dye negative.

### Flow Cytometery and Analysis of Cellular Phenotypes

BALF samples were spun at 210G for 8 minutes in a Sorval benchtop RT7 centrifuge. Cell pellets were resuspended in 100 µl Fc block to prevent non-specific antibody binding and incubated for 10 minutes on ice, subsequently samples underwent a 30-minute dark incubation with commercially available anti-mouse CD11b, CD11c, SiglecF, Ly6c, and Ly6G fluorophore-conjugated antibodies (BD Biosciences, BD Pharmingen, San Diego, CA). After a rinse step, samples were examined on FACSCantos, LSR II, or Acuri flow cytometers (Becton Dickinson, Mountain View, CA). Unstained cells from lavage samples collected at the highest infective dose used for each experiment were run to control for autofluorescence. Further data analysis and graph creation was performed with FlowJo software (Ashland, OR).

### Histology of Cytospin Preparations with Visualization by Microscopy

Aliquots (100 µl) were taken from resuspensions of FACS solutions fixed in ice cold EtOH for one hour in the dark and adhered to a slide via cytospin centrifugation (Shandon Cytospin). Cell surface staining for CD11c was achieved using a FITC rat anti-mouse CD11c antibody (BD Biosciences, BD Pharmingen, San Diego, CA). Bacteria were visualized either by fluorescent protein expression (GFP NMII) or by permanent red staining with *C. burnetii* as the target antigen (Dako North America, Inc., Carpinteria, CA). Slides were coverslipped with ProLong Gold antifade reagent with DAPI (Molecular Probes/Invitrogen, Eugene OR) and imaged at 40X on a Nikon E800 fluorescent scope (Nikon Instruments, Melville NY). Composite image overlays were made using NIS-Elements Nikon Image Analysis software.

### Cell Counts, Differentials and Total Cell Analysis

Total cells counts were obtained for BALF on a standard hemocytomer, using trypan blue exclusion of dead cells. Cytopsins stained with Diff-Quick (Siemens Healthcare Diagnostics, Newark DE) for differential analysis allowed for enumeration of various cell-types. Total cells for each individual type were obtained by multiplying total cell counts by the percentage for each individual type.

### Statistical Analysis

Prism Software (Prism Software Corporation, Orange County, CA) was used to produce all graphs and perform statistical analysis on data. All data (excluding FACS plots and photomicrographs) is expressed as log transform of change in bacterial burden. Graphs show mean and standard error of the mean (SEM) for all groups plotted. Student’s T test was run to test for statistically significant differences between paired groups, p<0.01 was considered significant. ANOVA was run to test for statistically significant differences between multiple groups, p<0.05 was considered significant.

## Supporting Information

Figure S1 **Lower doses of NMII resulted in greater relative bacterial numbers in the lungs of BALB/c Mice.** BALB/c mice were infected with either 10^3^, 10^5^ or 10^7^ NMII *Coxiella*. Bacterial burdens in lung tissue were assessed 2 (A), 9 (B), 16 (C) or 24 (D) days PI by quantitative RT-PCR. Data is expressed as Log10 change in total genome copies per lung (Endpoint bacterial genome copies per lung/Inoculum). Groups were comprised of 4–5 mice with Mean and SEM plotted for each group. Relative to all other inoculum concentrations, the 10^3^ group showed a significant increase in bacterial burden across the timecourse of infection. The 10^5^ group showed a significant initial increase in bacterial burden and a subsequent significant reduction in overall clearance of bacteria, relative to high dose infections. The 10^7^ group showed significant bacterial clearance relative to the other two doses from 9 days PI onward (ANOVA p<0.05).
(TIFF)

Figure S2 **High dose NMII infection did not result in increased clinical symptoms of systemic infection in BALB/c Mice.** A) BALB/c mice were infected with either 10^3^, 10^5^ or 10^7^ NMII *Coxiella*. Body weights were recorded daily from day 0 to day 24 PI. Groups were comprised of 4–5 mice with Mean and SEM plotted for each group. Although, there were small significant differences in final body weights among the three doses, none of the doses lost weight across the course of the infection. B) BALB/c mice were infected with either 10^3^, 10^5^ or 10^7^ NMII *Coxiella* and spleen weights were taken at each endpoint (days 2, 9, 16 and 24 PI). Groups were comprised of 4–5 mice with Mean spleen weight as a percentage of body weight and SEM plotted for each group. There are no significant differences between groups or across the timecourse of infection.
(TIFF)

Figure S3 **Lower doses of NMI resulted in greater relative bacterial numbers in the lungs of BALB/c Mice.** A) BALB/c mice were infected with either 10^3^, 10^5^ or 10^7^ NMI *Coxiella*. Bacterial burdens in lung tissue were assessed 2 (A), 9 (B), 16 (C) and 23 (D) days PI by quantitative RT-PCR. Data is expressed as Log10 change in total genome copies per lung. Groups were comprised of 4–5 mice with Mean and SEM plotted for each group. Relative to the other inoculum concentrations, the 10^3^ group showed a significant increase in bacterial burden from 9 days PI onward. The 10^5^ group showed a significant increase in bacterial burden, relative to high dose infections from 9 days PI onward. The 10^7^ group showed significant clearance relative to the other two doses from 16 days PI onward (ANOVA p<0.05).
(TIFF)

Figure S4 **Lower doses of NMI resulted in greater relative bacterial numbers in the spleen of BALB/c Mice.** A) BALB/c mice were infected with either 10^3^, 10^5^ or 10^7^ NMI *Coxiella*. Bacterial burdens in spleen tissue were assessed 2 (A), 9 (B), 16 (C) and 23 (D) days PI by quantitative RT-PCR. Data is expressed as Log10 change in total genome copies per spleen. Groups were comprised of 4–5 mice with Mean and SEM plotted for each group. Relative to the other inoculum concentrations, the 10^3^ group showed a significant increase in bacterial burden at 9 days PI. The 10^7^ group showed significant clearance relative to the other two doses from 9 days PI onward (ANOVA p<0.05).
(TIFF)
