# Supplementary figures and images for: Resident Alveolar Macrophages Are Susceptible to and Permissive of Coxiella burnetii Infection
Source: PLoS One. 2012 Dec 19;7(12):e51941. doi: 10.1371/journal.pone.0051941 (PMC3526480; doi:10.1371/journal.pone.0051941)

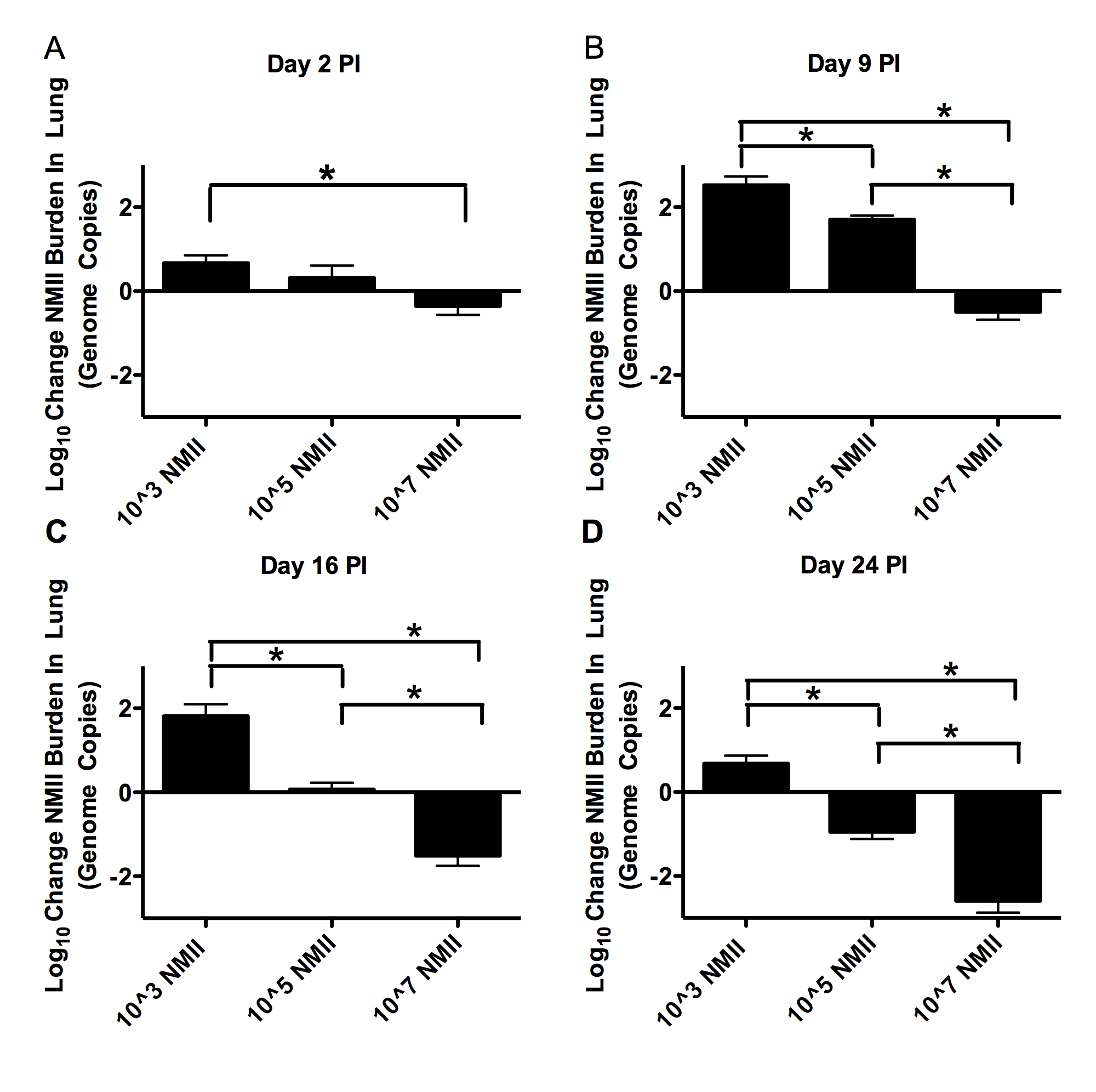

Supplement: Figure S1 — Lower doses of NMII resulted in greater relative bacterial numbers in the lungs of BALB/c Mice. BALB/c mice were infected with either 103, 105 or 107 NMII Coxiella. Bacterial burdens in lung tissue were assessed 2 (A), 9 (B), 16 (C) or 24 (D) days PI by quantitative RT-PCR. Data is expressed as Log10 change in total genome copies per lung (Endpoint bacterial genome copies per lung/Inoculum). Groups were comprised of 4–5 mice with Mean and SEM plotted for each group. Relative to all other inoculum concentrations, the 103 group showed a significant increase in bacterial burden across the timecourse of infection. The 105 group showed a significant initial increase in bacterial burden and a subsequent significant reduction in overall clearance of bacteria, relative to high dose infections. The 107 group showed significant bacterial clearance relative to the other two doses from 9 days PI onward (ANOVA p<0.05). (TIFF) [file pone.0051941.s001.tiff]

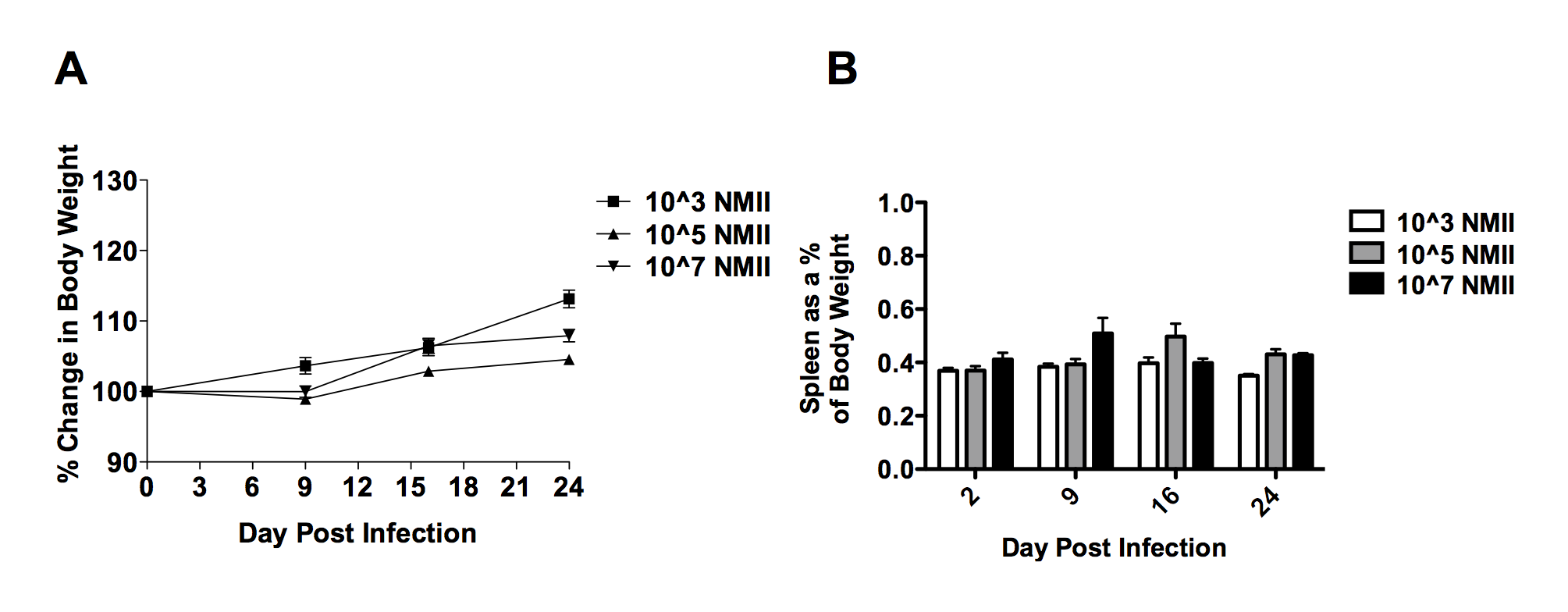

Supplement: Figure S2 — High dose NMII infection did not result in increased clinical symptoms of systemic infection in BALB/c Mice. A) BALB/c mice were infected with either 103, 105 or 107 NMII Coxiella. Body weights were recorded daily from day 0 to day 24 PI. Groups were comprised of 4–5 mice with Mean and SEM plotted for each group. Although, there were small significant differences in final body weights among the three doses, none of the doses lost weight across the course of the infection. B) BALB/c mice were infected with either 103, 105 or 107 NMII Coxiella and spleen weights were taken at each endpoint (days 2, 9, 16 and 24 PI). Groups were comprised of 4–5 mice with Mean spleen weight as a percentage of body weight and SEM plotted for each group. There are no significant differences between groups or across the timecourse of infection. (TIFF) [file pone.0051941.s002.tiff]

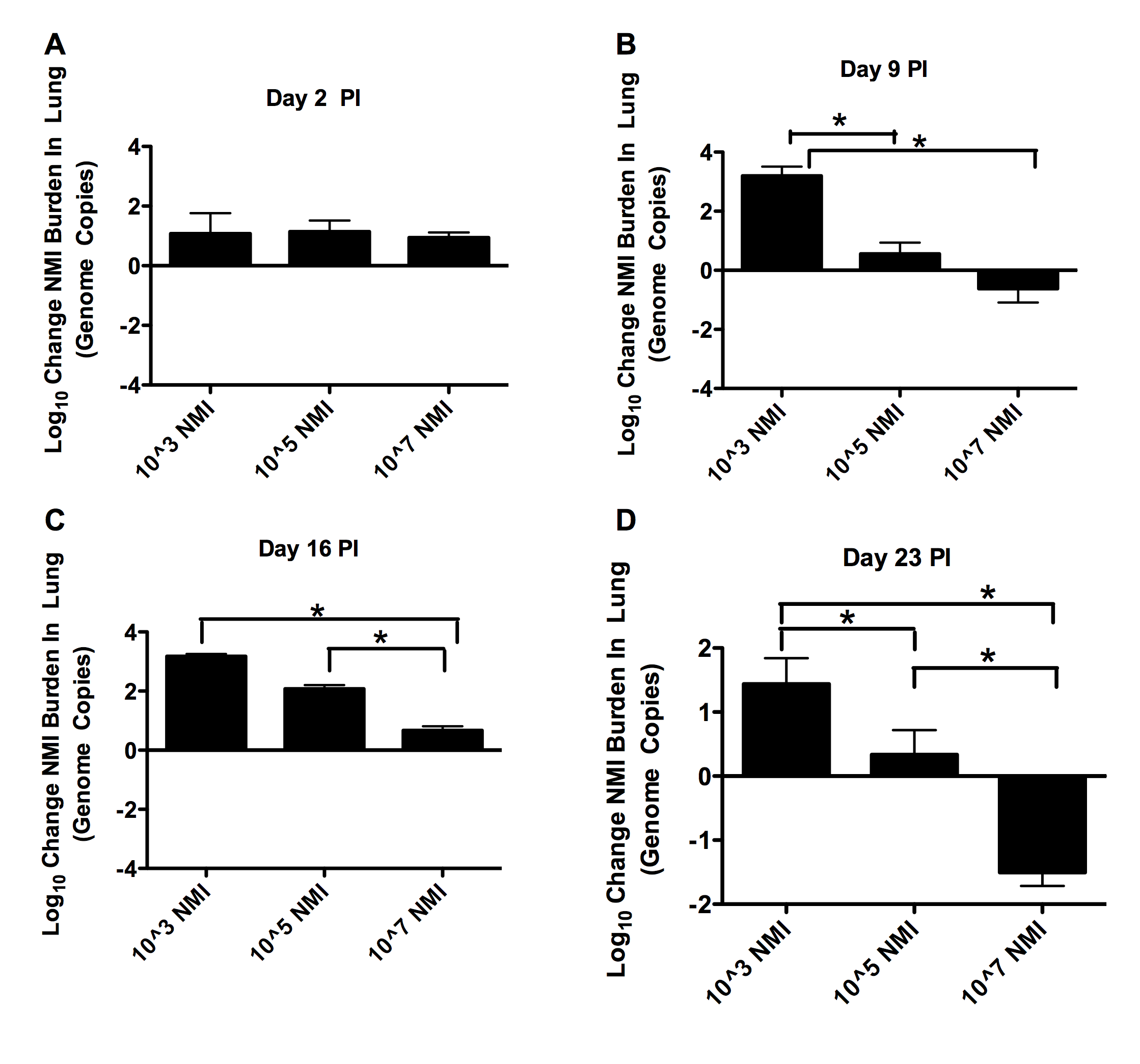

Supplement: Figure S3 — Lower doses of NMI resulted in greater relative bacterial numbers in the lungs of BALB/c Mice. A) BALB/c mice were infected with either 103, 105 or 107 NMI Coxiella. Bacterial burdens in lung tissue were assessed 2 (A), 9 (B), 16 (C) and 23 (D) days PI by quantitative RT-PCR. Data is expressed as Log10 change in total genome copies per lung. Groups were comprised of 4–5 mice with Mean and SEM plotted for each group. Relative to the other inoculum concentrations, the 103 group showed a significant increase in bacterial burden from 9 days PI onward. The 105 group showed a significant increase in bacterial burden, relative to high dose infections from 9 days PI onward. The 107 group showed significant clearance relative to the other two doses from 16 days PI onward (ANOVA p<0.05). (TIFF) [file pone.0051941.s003.tiff]

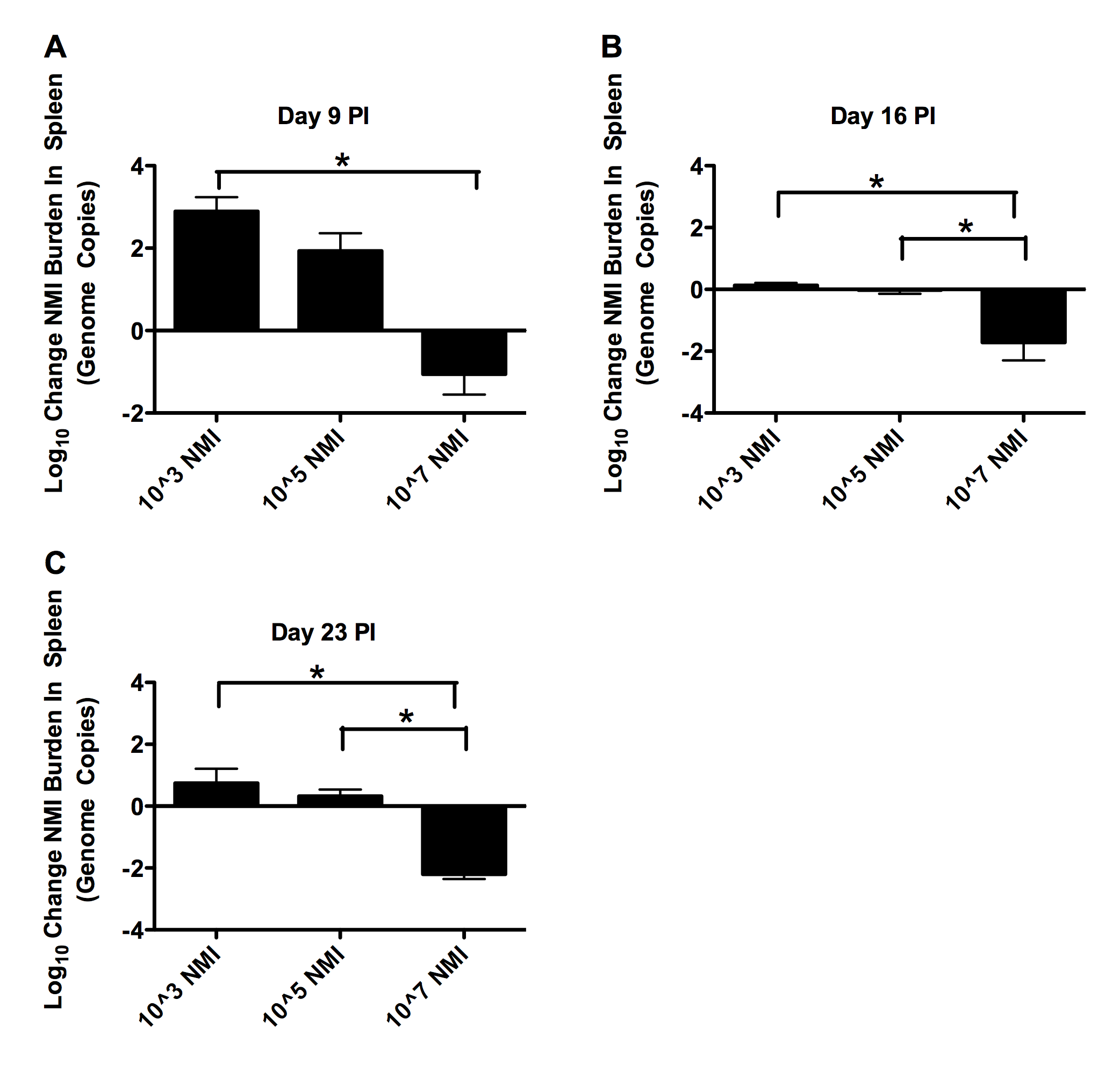

Supplement: Figure S4 — Lower doses of NMI resulted in greater relative bacterial numbers in the spleen of BALB/c Mice. A) BALB/c mice were infected with either 103, 105 or 107 NMI Coxiella. Bacterial burdens in spleen tissue were assessed 2 (A), 9 (B), 16 (C) and 23 (D) days PI by quantitative RT-PCR. Data is expressed as Log10 change in total genome copies per spleen. Groups were comprised of 4–5 mice with Mean and SEM plotted for each group. Relative to the other inoculum concentrations, the 103 group showed a significant increase in bacterial burden at 9 days PI. The 107 group showed significant clearance relative to the other two doses from 9 days PI onward (ANOVA p<0.05). (TIFF) [file pone.0051941.s004.tiff]
